# Identifying best approaches for engaging patients and family members in health informatics initiatives: a case study of the Group Priority Sort technique

**DOI:** 10.1186/s40900-020-00203-8

**Published:** 2020-05-18

**Authors:** Brian Lo, Timothy Zhang, Kevin Leung, Rohan Mehta, Craig Kuziemsky, Richard G. Booth, Anna Chyjek, Sarah Collins Rossetti, Drew McLean, Elizabeth Borycki, David McLay, Justin Noble, Shawn Carter, Gillian Strudwick

**Affiliations:** 1 Centre for Addiction and Mental Health, 1001 Queen Street West, Toronto, M6J 1H4 ON Canada; 2 Institute of Health Policy, Management and Evaluation, University of Toronto, 155 College Street, Toronto, M5T 1P8 ON Canada; 3 School of Public Health and Health Systems, University of Waterloo, 200 University Avenue West, Waterloo, N2L 3G1 ON Canada; 4 Telfer School of Management, University of Ottawa, 55 Laurier Avenue East, Ottawa, K1N 6N5 ON Canada; 5 Arthur Labatt Family School of Nursing, Western University, 1151 Richmond Street, London, N6A 3K7 ON Canada; 6 Department of Biomedical Informatics and School of Nursing, Columbia University, 622 W. 168th Street, Presbyterian Building 20th Floor, New York, 10032 NY USA; 7 McMaster University, 1280 Main Street West, Hamilton, L8S 4L8 ON Canada; 8 School of Health Information Science, University of Victoria, Human & Social Development Building A202, 3800 Finnerty Road (Ring Road), Victoria, V8P 5C2 BC Canada; 9 Canada Health Infoway, 150 King Street West Suite 1300, Toronto, M5H 1J9 ON Canada; 10 Ontario Shores Centre for Mental Health Sciences, 700 Gordon Street West, Whitby, L1N 5S9 ON Canada

**Keywords:** Nursing informatics, Patient engagement, Health information technology, Health informatics, Participatory research, Group priority sort

## Abstract

**Background:**

Patient engagement strategies in health service delivery have become more common in recent years. However, many healthcare organizations are challenged in identifying the best methods to engage patients in health information technology (IT) initiatives. Engaging with important stakeholders to identify effective opportunities can inform the development of a resource that addresses this issue and supports organizations in their endeavors. The purpose of this paper is to share our experience and lessons learned from applying a novel consensus-building technique in order to identify key elements for effective patient engagement in health IT initiatives. This will be done through a case study approach.

**Methods:**

Patients, family members of patients, health professionals, researchers, students, vendor representatives and individuals who work in health IT roles in health organizations were engaged through a one-day symposium in Toronto, Canada in September, 2018. During the symposium, the Group Priority Sort technique was used to obtain structured feedback from symposium attendees in the context of small group discussions. Descriptive statistics and a content analysis were undertaken to analyze the data collected through the Group Priority Sort as well as participant feedback following the symposium.

**Results:**

A total of 37 participants attended the symposium from a variety of settings and organizations. Using the Group Priority Sort technique, 30 topics were classified by priority to be included in a future resource. Participant feedback pertaining to the symposium and research methods was largely positive. Several areas of improvement, such as clarity of items, were identified from this case study.

**Conclusions:**

The Group Priority Sort technique was an efficient method for obtaining valuable suggestions from a diverse group of stakeholders, including patients and family members. The specific priorities and feedback obtained from the symposium will be incorporated into a resource for healthcare organizations to aid them in engaging patients in health IT initiatives. Additionally, five important considerations were identified when conducting future work with the Group Priority Sort technique and are outlined in this paper.

## Plain English summary

The success of health information technology initiatives, which comprises of making complex decisions related to the development, implementation and evaluation of health information technology (e.g., electronic medical records, patient portal), rests on engaging all relevant stakeholders including patients and family members. The purpose of this paper is to share our experience and lessons learned from applying a novel consensus-building technique to identify best approaches for meaningful patient engagement in these initiatives. The goal of this work was to develop a practical resource for healthcare organizations to use in their own health information technology projects. This study utilized a Group Priority Sort technique to gain a consensus of content to guide the development of a practical resource during a one-day symposium held in Toronto, Canada with 37 stakeholders. Participants included patients, family members of patients, health professionals, researchers, students, vendor representatives and people who work in health information technology roles in healthcare organizations. These individuals came from different geographical locations in the province of Ontario and held roles or had experiences in a diverse number of healthcare organizations. Conducting a Group Priority Sort in a symposium format paved the way for successful classification and ordering of 30 themes and topics by importance, as well as an engaged discussion on relevant topics such as knowledge translation and dissemination. In summary, using the Group Priority Sort technique to obtain structured feedback through the engagement activities described in this paper was an effective way of gaining insights from a number of diverse stakeholders.

## Background

Patient engagement is often cited as an essential approach to address the ongoing gaps caused by disparity in views, needs, and perceptions between stakeholders (e.g., physicians, patients, family members) [1]. As a result, patient engagement has become an increasingly important area of interest [2, 3] across all levels and areas of healthcare, from the co-development of a care plan for a specific individual [4] to the improvement of healthcare services [5, 6]. Studies on this topic have suggested that patient engagement is associated with various benefits including more effective healthcare delivery that is responsive to the needs and values of patients [7, 8] and the enhanced retention and dissemination of outcomes in healthcare research [5].

In response to the observed benefits, many guidance documents, tools, and frameworks have been developed on topics related to patient engagement for healthcare. Reviews on this topic in the context of health research have suggested that several degrees of engaging patients exist, and can range from the patient learning and informing a project to supporting and leading a project [7, 9]. Organizations such as the Patient-Centered Outcomes Research Institute (PCORI) in the United States have been established to advocate for standards (e.g., WE-ENACT tool) on how to best engage patients [10]. Likewise, the Canadian Institute for Health Research [11] has developed a patient engagement framework to support patient-centered research in Canada, and the Centre of Excellence on Partnership with Patients and the Public [12] released a toolkit to evaluate patient engagement activities in health care and research.

However, there remains a paucity of guidance on how to implement patient engagement in health IT initiatives, which includes making decisions related to the acquisition, development, implementation and evaluation of health IT (e.g., patient portals, mobile apps) and frequently forms the backbone of health IT success [13, 14]. While the uptake of health IT [15] has resulted in over $16 billion in estimated benefits to Canadians between 2007 to 2015 [16], in many cases healthcare organizations have not consistently taken full advantage of health IT [17]. As a result, many of their potential benefits to patients, families, and the healthcare system remain unrealized. Previous studies have identified that effective engagement of patients and family members throughout the activities (e.g., selection, adoption, evaluation) of health IT initiatives can generate improved outcomes [18–20] and maximize the return on investment of these technologies [21]. This need is particularly important given the increasing demand of patients and family members to be part of healthcare initiatives [22]. Other areas of concern include how to empower these important stakeholders throughout the engagement process [23] and how to define and evaluate success of patient engagement efforts for patients and healthcare organizations [9], particularly in environments with limited time and resources.

In order to generate this guidance document, this project sought the advice of a diverse set of stakeholders. One technique that can be used to engage and gather useful feedback from multiple stakeholders, including patients and family members, is the Group Priority Sort technique [24]. The Group Priority Sort is a structured technique originally developed by Jacobson and colleagues [24] to engage participants and collect feedback and information for decision making by healthcare leaders. This technique is particularly helpful in situations where items of importance are required from a diverse number of stakeholders (e.g., healthcare administrators, clinicians, patients) [24]. This technique has been applied in various situations including identifying areas of priority in inter-professional education [24], developing a Canadian competency framework for addictions [25], and exploring areas of focus for substance abuse [26]. The Group Priority Sort [24] is comprised of two steps: a group rapid-sort to provide initial identification of useful and important content, followed by a forced-choice sort, where consensus on items is reached through discussion.

The purpose of this paper is to share our experience and lessons learned in a case study format from applying a novel consensus-building technique (Group Priority Sort technique) to identify best approaches for meaningful patient engagement in health IT initiatives. Specifically, we engaged relevant stakeholders to identify best approaches to engage patients and family members in health IT initiatives and inform the development of a resource to support organizations through such work [27]. By presenting this case study, we demonstrate the feasibility and robust utility of the Group Priority Sort method for consensus gaining, and share our lessons learned to identify key areas of consideration for future applications of this approach.

## Methods

The activities described in this paper were completed at a one-day symposium at a large academic hospital located in Toronto, Canada in September of 2018. Further details related to the symposium are discussed in the following sections.

### Planning and development of symposium

The planning and development of the symposium was completed in close collaboration with four patients and family members. One of the authors of this paper (RM) is a patient representative who participated in all aspects of this research project including, but not limited to, brainstorming, logistics planning, findings interpretation and manuscript submission. In addition, a Patient and Family Advisory Council, which consisted of four members (two patients and two family members), provided substantial input throughout the planning stage at monthly meetings with the members of the research team. This took place over the course of 6 months prior to the event. After the event, there were two debrief meetings that took place.

### Symposium structure

The goal of the symposium was to reach a consensus on essential elements for the guidance document on how to best engage patients and family members in health IT initiatives, as well as identify potential knowledge translation strategies to disseminate these findings to healthcare organizations. The symposium began with a keynote speech on the topic of ‘Patient Engagement in Health Informatics Research’ followed by a panel talk representing patients, family members, and health professionals’ experiences on collaboratively engaging in health IT initiatives. To further ensure all participants had at least a baseline understanding of the symposium’s research activities, another presentation on the results of a literature review [28] and findings from focus groups with patients on the topic of patient engagement methods in health IT initiatives was delivered. The two focus groups, which were conducted prior to the symposium, surveyed the perspectives of patients and family members on patient engagement in healthcare in general, and strategies frequently used in these initiatives [27]. In the afternoon, the Group Priority Sort technique was used to obtain structured feedback from the participants in small groups. The content of the Group Priority Sort was informed by previous focus groups with patients, and feedback from patients and family members. At the end of the day, participants were asked to fill in a paper survey to evaluate their experience participating in the symposium. Ethical approval was obtained from the Centre for Addiction and Mental Health Research Ethics Board (004/2018).

### Participants and recruitment

To recruit participants with diverse backgrounds and perspectives, targeted email invitations were sent to health professionals, researchers, vendor representatives, patients, family members of patients, students, and knowledge users (e.g., informatics professionals employed at a healthcare organization in Ontario). Approximately 6–8 invitations were sent to each of these user groups. Recruitment continued through a ‘snowball’ technique where members of the Patient and Family Advisory Council at the main study site and invited recipients were encouraged to forward the invite to other individuals who may be interested in participating in the symposium. Given that a ‘snowball’ technique was used for recruitment, it is unknown how many participants were invited in total. Additionally, recruitment activities included posting of recruitment material on an organizational website and an organizational research portal, and advertising in a newsletter through the Office of Family Engagement. Eligibility for participation included being a member of one of the previously mentioned participant groups and having some prior knowledge of the topic. For those participants who were invited to the event via email, conflicting schedules and prior commitments were the most common reasons given for being unable to attend.

A total of 37 individuals attended the symposium. Specifically, the participants were comprised of 4 patients, 2 family members of patients, 9 graduate students, 2 health informatics researchers, 9 health professionals, 2 health IT vendor representatives, and 9 knowledge users (e.g., management staff, project analysts). Participants worked at, or had experience at, a variety of healthcare organizations from various urban and rural settings in Ontario, Canada.

### Group Priority Sort

This process was used to reach consensus within small, diverse groups of 4–6 participants in a manner which was highly relevant given the diverse background and role (e.g., knowledge user, patients) of stakeholders in health IT initiatives and the subjective nature of this research. This method allows for rich discussions to take place to shape how consensus is gained. In the following sections, the steps that were taken to complete the group priority sort technique are described.

#### Step 1: Group rapid-sort

To begin the group rapid-sort process, participants were asked to divide into groups of 4–6. To ensure diversity within each group, a pseudo single-blind process was used where each participant was provided with a colored sticker based on their stakeholder type (e.g. patient, health professional). The participants were then instructed to form groups with a diverse set of sticker colors, unaware of the color’s purpose. A facilitator and a note-taker were randomly assigned to each group and the former presented the group with a deck of 30 pre-populated index cards. Each card displayed a unique item (potential content for the resource), which had been identified through a preceding literature review and focus groups. Some of the items were slightly modified by the research team to ensure they could be adequately understood and assessed at face value by all participants. Facilitators then asked the group of participants to individually rate the first item on a Likert scale from 1 to 5 (1 being the least important, and 5 being the most important) on how the given item should be prioritized as a topic to be included in the resource that would be developed. The participants were encouraged to give a rating quickly with little consideration in an effort to capture their instinctive judgement. Following the 30 items, participants were asked to brainstorm any items which they believed to be missing. Any new items were recorded and rated by the group. Participants’ ratings for all 30 items, in addition to those added by the group, were recorded by note-takers. The main goal of this step was to provide a baseline for the last phase of the group priority sort technique which is called ‘group-forced sort’ and requires the group to come to consensus on their priorities [24].

#### Step 2: Group brainstorming

In the same groups, participants were asked to brainstorm potential methods and outlets for knowledge translation and dissemination of the resource, as well as how the final product should be formatted and/or structured (e.g. how it should look, how it should be accessed). This portion of the group activity was included as an additional part of the symposium’s research and not part of the Group Priority Sort. Participants were asked to be as descriptive and detailed as possible when making these suggestions. Note-takers captured the discussion points on white-paper.

#### Step 3: Group forced-sort

The group forced-sort required consensus within each group. To facilitate this, all index cards from the ‘Group Rapid Sort’ phase were presented back to the group marked with the participant ratings for reference. The participants were then asked to sort all the items into a 5-point Likert scale as a group, and encouraged to discuss amongst themselves to do so. They were instructed that each Likert scale point, or ‘category’, had to contain at least 6 but not more than 7 (because participants could have added items to the original 30). Both the facilitator and note-taker (who are not participants) were actively present to aid the participants in thinking critically about their priorities while ensuring all opinions were being heard in achieving consensus. The main goal achieved through this activity was to identify the relative priority of each item compared to the rest of the items in the set through strict parameters [24]. At the end of group forced-sort activities, groups presented back to the broader group to share their rankings as well as thought process and rationale for their rankings.

### Symposium evaluation

In order to gain insight into the experience, needs and perceptions of participants, a brief evaluation was conducted following the symposium. Participants completed a paper survey to provide feedback on the symposium by rating elements on a 7-point Likert scale (e.g., venue, speakers, refreshments, research activities). This evaluation was followed by a second electronic survey sent out 3 days after the symposium by email through an online survey platform, which asked participants to respond to open-ended questions on the research activities and obtain their overall impression of the symposium once they had a chance to reflect upon it. Quantitative data was analyzed using descriptive measures, and qualitative analysis on open-ended responses was conducted using content analysis [29].

## Results

### Group Priority Sort results

The Group Priority Sort technique enabled participants to come to a consensus on the relative importance of the given 30 items, effectively achieving the goals of this research activity. After each group sorted their items into the five-point Likert-scale categories, the average score for each item across all groups was used to arrange the 30 items into a table. A clear distinction emerged between the six most and four least important items (Table 1), while the remaining items fell somewhere in-between. A full summary of the symposium’s research activity results can be found here [27].

**Table 1:** Overview of most and least important items identified from Group Priority Sort exercise [27]

Most Important (5.00–4.00)	• Recognition of power dynamics • Governance structures • Engagement in HIT evaluation • Engagement in requirements gathering processes • Engagement training for health care staff • Patient and family HIT training
Least Important (1.99–1.00)	• List of Ontario patient and family engagement network groups • Literature review describing benefits of patient and family engagement in HIT • Food and refreshments during meetings • List of non-HIT-related patient and family engagement resources

### Participant feedback results

Participants were given two opportunities to provide feedback since some participants may have wanted to reflect on the symposium before responding. One method was a paper survey at the conclusion of the symposium, and the other was a follow-up online survey. The majority (96%) of participants (*n* = 25 of 26) completed the paper survey at the conclusion of the symposium (only 26 received the paper survey as some participants had left before it was handed out), and 48% of participants (*n* = 18 of 37) completed the second follow-up online survey. Feedback surrounding all aspects of the symposium was overall positive. Participants were very satisfied with the venue, date/time, and knowledge dissemination presentations (overall mean of 6.4 on all 1–7 Likert scale questions). The speakers in the first part of the symposium were very well received (overall mean of 6.3/7 Likert scale). Participants found the talks informative, interesting, and fit into the event well, with one participant noting:

*“I really appreciated the mix of speakers in the morning (which was about sharing knowledge with participants) and activity in the afternoon (which was participants sharing knowledge with the team). A very equitable way to work. Thanks.”*

With regards to questions surrounding the research activities, all responding participants felt they were both able to meaningfully contribute to the research and felt comfortable doing so. All respondents also reported that they felt they had enough knowledge on the topic of health IT and patient/family engagement to partake in the research activity.

*“Everything was very active; the group activity allowed for everyone to speak and participate”*

*“Good energy, presentation moved along well, interactive; won't change format”*

Only two participants in the online survey found that the Group Priority Sort technique was not effective for gaining consensus. Feedback related to the clarity of the instructions for the technique were mixed, but emphasized the importance of facilitators overseeing and guiding each group:

*“The instructions were clear and I understood what was asked of me which made it easy to do the activity”*

*“There were quite a bit of instructions for the activity but having a facilitator really helped get the ball rolling … ”*

*“Clear, instructions with helpful moderators; answered all the questions I had”*

*“Complex instruction, but facilitator assisted with clarification”*

In response to the question: “Is there anything you would change about this activity to make it easier for you to participate?”, some participants noted that a point of friction was the clarity of the given 30 items. As mentioned above, these 30 items were items or topics of interest identified through a previous literature review and focus groups [27, 28], and then re-worded for conciseness for the purposes of this research activity. While a facilitator was available to provide explanations of each item, a few participants noted that there was some additional discussion surrounding interpreting the meaning of some theme items, and perhaps supplementary explanations or definitions of the terms could be provided on paper to enhance clarity throughout the activity.

*“ … would have been great to clearly define some of the cards (e.g., governance structure, etc.)”*

*“... many of the cards were unclear as to what they really referred to, leaving much to interpretation which, as was clear in the group feedback, will make it hard to get as clear a direction from the group as you probably hoped.”*

Additional open-ended comments by participants again commended the quality of the speakers and the symposium as a whole, and expressed gratitude for the networking opportunity and ability to have a voice.

## Discussion

The current case study examines a novel approach for gaining consensus on how to best engage patients and family members and identify priority areas for health IT initiatives within a one-day session symposium. The use of the Group Priority Sort [24] in this case study illustrates the feasibility and ability of this approach to capitalize on preliminary findings obtained through a literature review [28] and focus groups [27], and engage a large, diverse number of stakeholders on complex and difficult to define matters such as patient engagement. As observed from the end-of-symposium survey, many participants felt that the symposium was enjoyable and that the development of a resource document from the findings of the symposium would be considered a significant and important contribution to the field of health IT. In addition, combining the exercise with introductory activities, namely a keynote speech, panel discussion, and presentation on previous work, provided sufficient context for participants in completing the activity. Many of these findings were also supported by previous studies that used this exercise [25, 26]. The findings from this symposium have not just reiterated the need for guidance on patient engagement strategies for health IT initiatives, but have also shown the changes needed at the systems level to enhance capacity and potential for patient engagement in these initiatives. In particular, these include developing the proper governance structures for patient engagement and equipping patients and family members with the necessary knowledge around digital health literacy [30] to actively participate in health IT initiatives [27].

Our case study with using the Group Priority Sort for a complex healthcare topic highlights several key factors (Table 2) that are critical for the success of this activity, which supports previous work [24, 26]. Foremost, participation from a diverse group of stakeholders (i.e., industry, academia and healthcare organizations) was instrumental in gaining comprehensive feedback for the topic. As a result, to ensure that feedback is fulsome and representative, a thoughtful sampling strategy is needed to engage a wide range of stakeholders in completing the activity. Another success factor was the selection of presentations and guest speakers prior to the Group Priority Sort activity. As identified in another study [26], the importance of the preceding activities (i.e., the keynote speaker and panel speakers) ensured the setting of the context and a common understanding going into the Group Priority Sort activities. As such, including relevant preceding activities such as keynote speaker topics may also ensure that all participants have a common understanding of the items and topics to be discussed and expectations prior to the activity.

**Table 2:** 5 key considerations on using Group Priority Sort to gain consensus from stakeholders

1. Ensure that items are clear and unambiguous by conducting pilot tests of items with sample of similar stakeholders.	
2. Ensure there is diversity in stakeholders (i.e., industry, academia, and healthcare organizations) in the activity for comprehensive and representative feedback	
3. Foster a safe environment for discussion to ensure that the voices of all stakeholders are heard (e.g., training of facilitators)	
4. Foster a common understanding of knowledge and context through strategic selection of activities (e.g., panel speakers) preceding Group Priority Sort exercise	
5. Leverage project management tools and methodologies to ensure successful planning and delivery of event in limited time and resources	

The current case study also identifies several areas of recommendations when conducting a Group Priority Sort activity [24]. Foremost, we reiterate the importance of ensuring that the items that are being ranked are clear and tangible to ensure that participants have a shared understanding of the items that are being ranked. To enhance clarity of each item, conducting a pilot test of the items among a sample of similar stakeholders may help address and reduce potential confusion with interpretation of each item during the activity. Second, given the differential levels of expertise and experience of the stakeholders, fostering a safe environment for discussion is essential to ensuring that the voices of all stakeholders are heard. This may be achieved through appropriate training of facilitators to promote an open and inviting environment for discussion [24]. Finally, the numerous activities that are held within the constraints of time and resources require proper planning and coordination. Leveraging project management skills can be useful in facilitating the planning and execution of the different components of the event.

This technique can be contrasted to other methodologies such as the Delphi technique [31] and the Nominal Group technique [32]. While the Delphi technique [31] is frequently used to obtain items of consensus from a large number of stakeholders, the Group Priority Sort [24] allows for the obtainment of context and rationale regarding how items were selected as often a discussion between participants ensues. However, while the Delphi technique can be conducted remotely (e.g., online surveys), the Group Priority Sort technique is relatively more resource intensive, requiring a higher level of commitment from participants given its in-person and single-session nature. Likewise, the Group Priority Sort is similar to the Nominal Group technique as it fosters discussion among the group on identifying consensus on a specific issue or problem [32]. However, the Nominal Group technique [33] requires that stakeholders brainstorm potential items for prioritization and does not typically include a component for discussion on the rationale for prioritization. Similarly, the Group Priority Sort technique [24] requires items to be identified in advance, and the success of the activity may be dependent on the robustness and quality of both the item identification process and the items themselves. The appropriateness of a chosen technique thus depends on a variety of factors including resources availability and the nature of the topic being studied.

In terms of results, the Group Priority Sort technique have also been beneficial in providing further context on the present priorities and concerns that remain in patient engagement locally in Canada. This is evident through the inclusion of additional items that were identified outside the literature review. For example, as healthcare organizations and provinces begin to form patient and family advisory councils [34], establishing proper governance structures that recognize and mitigate power dynamics would be of value. The collective identification of these needs from local stakeholders encourage local ownership of the findings which can be useful in encouraging the translation of results to practice [35].

### Implications

The findings from this case study demonstrate the feasibility and utility of the Group Priority Sort technique for both healthcare organization and health system researchers to engage participants for consensus-building. In particular, the key considerations identified from this case study supports the planning and use of the Group Priority Sort exercise to gain consensus on the prioritization of items within complex topics from a large number of stakeholders. In particular, when appropriate, this activity can be used by healthcare organizations alongside conferences and think tanks to systematically collect feedback and identify areas that are of importance.

In addition, the evidence base of this case study can be used to develop a toolkit to assist healthcare organizations with engaging patients and families in their own health IT initiatives. Through application and usage of the toolkit by healthcare organizations, feedback can be collected to help inform and refine future iterations of the toolkit.

### Future directions

The findings from this study provide several areas of further exploration. While the Group Priority Sort has been used by several studies, there remains a lack of robust evaluation of the strengths and weakness of this approach. As such, an in-depth evaluation on how this technique compares to other established methods (e.g., Delphi technique) is warranted. While we have identified 5 key areas of consideration, the paucity of guidance on the Group Priority Sort highlights the need to develop established guideline for other researchers to apply to their own projects and initiatives [25, 26]. Further refinement of the 5 areas of consideration using additional case studies is also warranted. The findings of this study have also identified the need to examine the dynamics of engagement throughout the Group Priority Sort. Future studies may wish to capture the different prioritizations, perspectives and feedback by different stakeholder groups, as well as the group dynamics throughout the engagement activity. This information may be useful in explaining and providing further context on the study findings.

## Conclusion

This case study demonstrates the feasibility and utility of the Group Activity Sort to gain consensus on an emerging and complex healthcare topic, which is how to effectively engage patients and family members in health IT initiatives. Through the engagement of relevant stakeholders, content of priority can be rapidly identified from a number of items [24]. This technique was effective in meeting our goal of identifying ways to effectively engage patients and family members in health IT initiatives. By ensuring that the five key success factors are addressed, the group priority sort can be another useful tool for researchers and health system administrators to gain consensus on complex and novel healthcare topics.

## Data Availability

Any additional materials may be available from the corresponding author upon reasonable request.
